# Extragenital *Mycoplasma hominis* infection following obinutuzumab therapy in a patient with chronic lymphocytic leukemia

**DOI:** 10.1016/j.idcr.2026.e02744

**Published:** 2026-09-03

**Authors:** Katherine Benandi, Lucas S. Blanton

**Affiliations:** a University of Texas Medical Branch, John Sealy School of Medicine, 301 University Blvd, Galveston, TX, 77555, United States; b Department of Internal Medicine, Division of Infectious Diseases, University of Texas Medical Branch, 301 University Blvd, Galveston, TX, 77555, United States

**Keywords:** *Mycoplasma hominis*, Septic arthritis, Obinutuzumab, Rituximab, Anti-CD20 monoclonal antibodies

## Abstract

*Mycoplasma hominis* is a fastidious, cell wall-deficient bacterium that typically colonizes the genitourinary tract but can cause invasive extragenital infections in immunocompromised hosts. Such infections have been reported primarily in patients receiving rituximab; however, they have not been previously described with obinutuzumab therapy. We report a case of *M. hominis* septic arthritis of the shoulder in a 70-year-old man with chronic lymphocytic leukemia/small lymphocytic lymphoma treated with Obinutuzumab. The patient presented with several months of progressive shoulder pain and radiographic evidence of rapidly destructive arthritis. Repeated joint aspirations and early cultures were negative, and he required multiple surgical washouts because of extensive purulence and periarticular abscess formation. Extended incubation of synovial fluid and tissue cultures ultimately identified *M. hominis*. Antimicrobial therapy was transitioned to levofloxacin and doxycycline, which were administered for six weeks with good tolerance and sustained clinical improvement. At follow-up, the patient remained free of recurrent infection with gradual recovery of shoulder function. This case highlights *M. hominis* as an emerging opportunistic pathogen in patients receiving newer anti-CD20 monoclonal antibodies and expands the recognized infectious complications of obinutuzumab-induced B-cell depletion. Because *M. hominis* lacks a cell wall, routine diagnostic methods and empiric β-lactam therapy may be ineffective, resulting in diagnostic delays. Awareness of this organism and early consideration of targeted therapy are critical to improving outcomes in immunocompromised patients.

## Introduction

*Mycoplasma hominis* is a fastidious, cell wall-deficient bacterium that typically colonizes the genitourinary tract but can cause invasive extragenital infections in immunocompromised hosts [1,2]. Its lack of a peptidoglycan cell wall confers intrinsic resistance to β-lactam antibiotics and contributes to diagnostic delays when relying solely on routine culture media. Extragenital *M. hominis* infections have been reported in a limited number of immunocompromised patients with both congenital and acquired causes of immune dysfunction. Most drug-related cases have occurred in the setting of rituximab-induced hypogammaglobulinemia, with reported presentations including septic arthritis, soft tissue abscesses, central nervous system infection, and disseminated disease [[3], [4], [5], [6], [7], [8], [9]]. B-cell-depleting monoclonal antibodies targeting CD20, such as rituximab and obinutuzumab, are increasingly utilized in the treatment of hematologic malignancies and autoimmune disorders. These agents lead to prolonged B-cell depletion and secondary hypogammaglobulinemia, predisposing to opportunistic infections, including viral reactivations and atypical bacterial or fungal pathogens [2,10].

Although extragenital *M. hominis* infections have emerged as a complication of rituximab therapy, our review of the literature revealed no prior reports of *M. hominis* infection occurring in the context of immunosuppression from obinutuzumab, a glycoengineered type II anti-CD20 monoclonal antibody [11]. We describe a case of *M. hominis* septic arthritis of the shoulder in a patient treated with obinutuzumab, highlighting the expanding infectious spectrum associated with next-generation B-cell-targeted therapies.

## Case presentation

A 70-year-old man with chronic lymphocytic leukemia (CLL)/small lymphocytic lymphoma (SLL) receiving obinutuzumab, whose cancer course was complicated by intermittent pancytopenia, and with a history of metastatic seminoma, presented with progressive left shoulder pain and swelling. The patient reported three months of worsening left shoulder pain beginning after a corticosteroid injection. An arthrocentesis performed two months before presentation showed no organisms or crystals. The patient was evaluated in the pain clinic the day before hospital admission. Radiographs following that appointment demonstrated cortical erosions concerning for septic arthritis, prompting urgent evaluation and hospital admission.

Additional medical history included a right orchiectomy and hemiscrotectomy for seminoma approximately four months prior to this admission. This surgery was complicated by an *Enterobacter cloacae* wound infection, which resolved after surgical debridement and 10 days of ertapenem. He also had essential hypertension, paroxysmal atrial fibrillation, and hypercholesterolemia. He denied tobacco use and reported minimal alcohol intake. Family history was notable for esophageal cancer in his father.

**Fig. 1:**
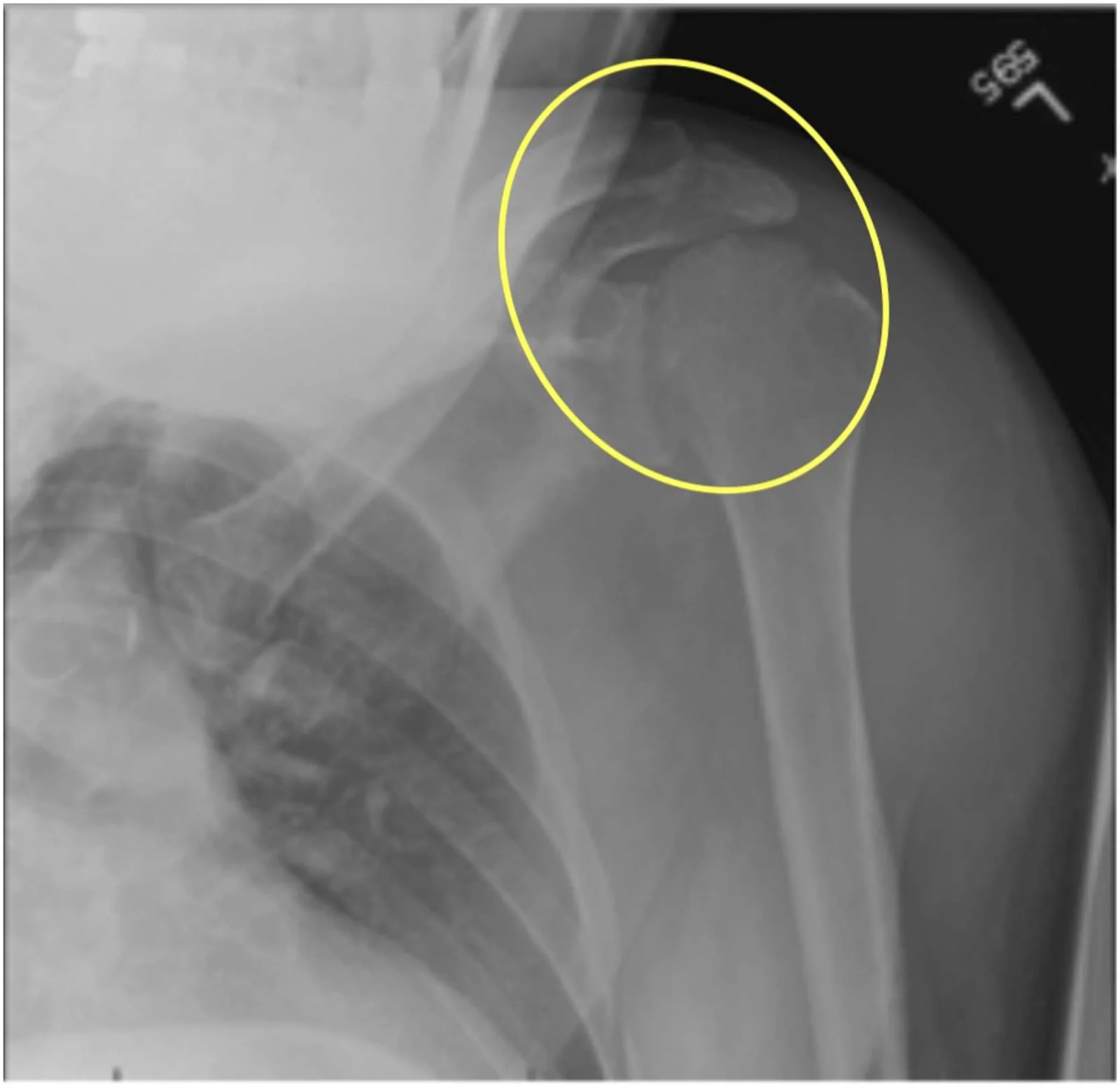
X-ray of left glenohumeral joint showing marked joint space narrowing with osteolysis of the humeral head and glenoid, and fragmentation at the inferior glenohumeral ligament.

**Fig. 2:**
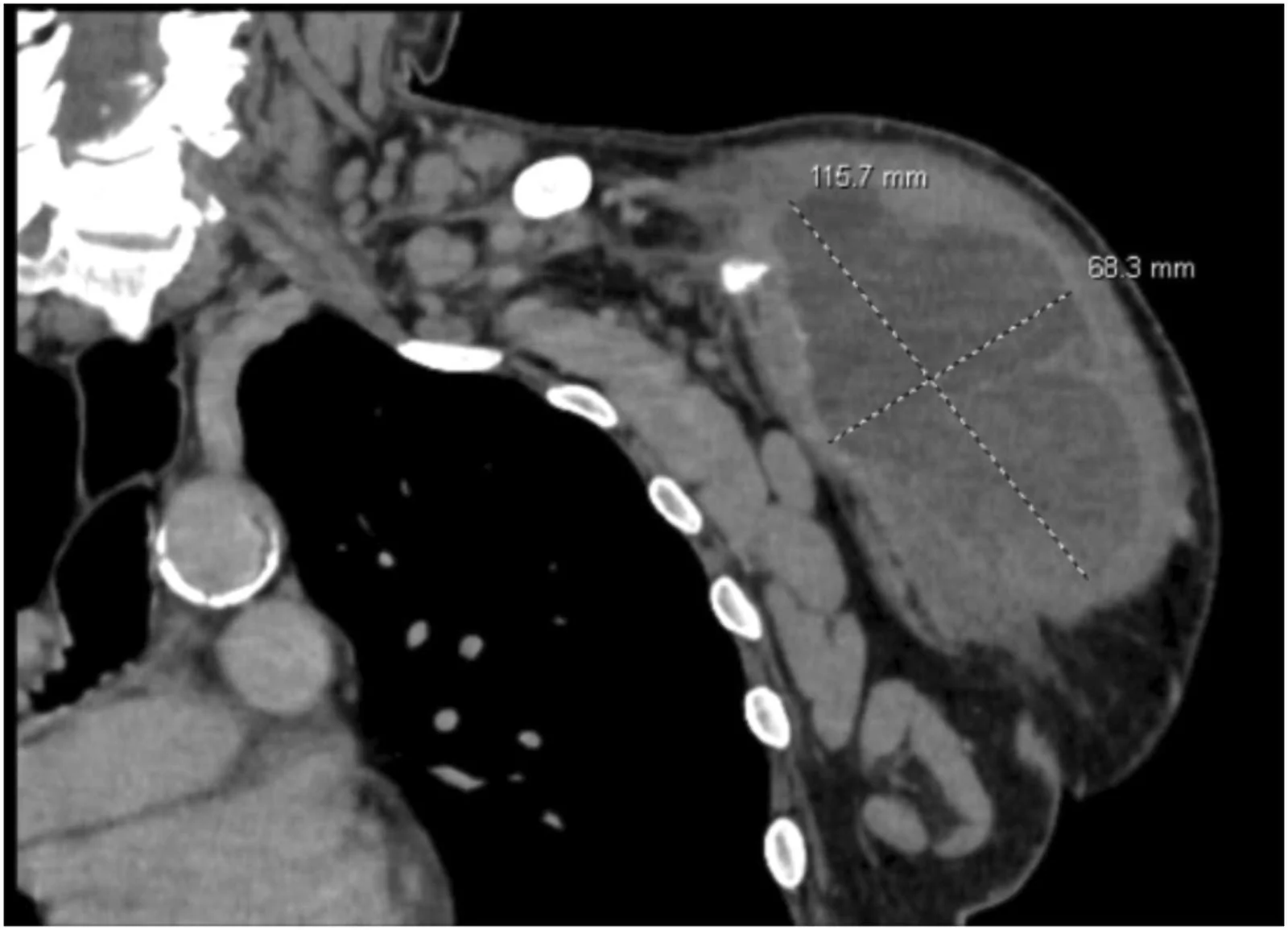
Contrast-enhanced CT of the left shoulder demonstrating a moderate glenohumeral joint effusion with severe joint space narrowing and cortical erosion of the humeral head and inferior glenoid, consistent with septic arthritis. A complex multiloculated, peripherally enhancing fluid collection extends into the deltoid, triceps, and supraspinatus muscles, compatible with intramuscular abscesses. Diffuse edema of the rotator cuff musculature is also present.

At the time of hospital presentation, his temperature was 37.3°C, blood pressure 138/68 mm of Hg, heart rate 97 beats/minute, and respiratory rate 18 breaths/minute. Examination of the left shoulder revealed tenderness to palpation and markedly decreased range of motion, without erythema, warmth, or drainage tracts**.** The remainder of the physical exam was otherwise unremarkable. Initial laboratory testing revealed a leukocyte count of 4500 cells/µL, hemoglobin of 10.2 g/dL, platelet count of 173,000/µL, serum creatinine of 1.05 mg/dL, C-reactive protein of 9.3 mg/dL, and an erythrocyte sedimentation rate of 17 mm/hour. An X-ray of the left shoulder demonstrated clear progression of disease, with new glenohumeral joint space narrowing and increasing osteolysis of the humeral head and glenoid compared to earlier imaging (Fig. 1). A subsequent CT scan demonstrated a large glenohumeral joint effusion, extensive cortical erosions, and multiloculated periarticular abscesses, suggestive of a rapidly destructive infectious process (Fig. 2). Empirical antimicrobial therapy was initiated, consisting of intravenous (IV) ceftriaxone 2 g every 24 h and IV vancomycin 1250 mg every 12 h. Prophylactic sulfamethoxazole and trimethoprim were discontinued, as they were deemed unnecessary while the patient was on IV antibiotics.

A repeat joint aspiration was performed upon admission, yielding turbid synovial fluid with a white blood cell count of 92,500 cells/µL (predominantly neutrophils), but the Gram stain showed no organisms. Based on these findings, the patient underwent surgical washout and debridement. During exploration of the shoulder, approximately 400 mL of purulent fluid was evacuated. Because of the extensive findings at the original surgery, an additional two surgical washouts were performed one and two weeks after admission, respectively. Despite abundant purulence noted during the first surgical procedure, all early cultures remained negative, raising concern for a fastidious organism.

Synovial fluid and operative tissue specimens were submitted for bacterial culture and inoculated onto routine aerobic and anaerobic solid media, including blood agar, chocolate agar, MacConkey agar, Colistin-Nalidixic Acid (CAN) agar, Brucella blood agar, and Bacteroides Bile Esculin (BBE) and Laked Blood Kanamycin Vancomycin (LKV) agar. Initial Gram stains of tissue specimens demonstrated no organisms, with only rare inflammatory cells observed. Because of ongoing clinical suspicion for infection, cultures were incubated for an extended fourteen-day period. After 4 days of incubation, minute pinpoint colonies were observed on Brucella blood agar and LKV agar, with subsequent growth appearing the following day on blood agar, chocolate agar, and CNA agar. The colonies remained too small to exhibit the characteristic “fried-egg” morphology typically associated with *Mycoplasma* species. Identification by matrix-assisted laser desorption/ionization time-of-flight mass spectrometry revealed an organism most consistent with *M. hominis*. The organism was recovered from synovial fluid and from multiple independently collected operative tissue specimens, supporting the diagnosis of *M. hominis* septic arthritis with associated osteomyelitis. Growth was scant, and the isolate could not be recovered on subsequent subculture. Consequently, antimicrobial susceptibility testing and molecular confirmation by polymerase chain reaction (PCR) could not be performed.

Following identification of *M. hominis*, empiric IV vancomycin and ceftriaxone were discontinued after seven days of therapy, and treatment was transitioned to oral levofloxacin 750 mg once daily and doxycycline 100 mg twice daily. The patient completed a six-week course of this regimen without adverse effects. At follow-up approximately two and six months after completion of therapy, he remained afebrile and free of recurrent infection, with continued improvement in pain and shoulder function. Although mild limitations in range of motion persisted, he reported substantial functional recovery and was again able to perform activities of daily living independently. No recurrence was observed despite subsequent episodes of neutropenia during treatment for his seminoma.

## Discussion

This case highlights *M. hominis* as an opportunistic pathogen causing extragenital infection in a patient with CLL/SLL receiving obinutuzumab, a type II anti-CD20 monoclonal antibody. The temporal association between B-cell depletion and infection supports humoral immunodeficiency as the major predisposing factor. Extragenital *M. hominis* infections in the setting of acquired immunosuppression have previously been reported almost exclusively in patients treated with rituximab, where profound B-cell depletion and hypogammaglobulinemia predisposed to septic arthritis, abscess formation, and disseminated disease [[3], [4], [5], [6], [7], [8], [9]]. The present case is distinct in that it occurred during obinutuzumab therapy, thereby expanding the recognized spectrum of infectious complications associated with newer anti-CD20 agents. Compared with rituximab, obinutuzumab is a newer-generation, glycoengineered type II anti-CD20 monoclonal antibody that induces more potent antibody-dependent cytotoxicity and deeper, longer-lasting B-cell depletion [11]. This may further extend the window of vulnerability to opportunistic infections, including atypical bacteria such as *M. hominis*.

The source of *M. hominis* infection in this case remains uncertain. Because *M. hominis* is a common colonizer of the genitourinary tract, hematogenous dissemination from this reservoir is the most likely mechanism of infection, particularly in the setting of obinutuzumab-induced humoral immunodeficiency and hypogammaglobulinemia [[12], [13], [14]]. It should be noted that the patient had undergone recent genitourinary surgery. This procedure, and/or urethral irritation from periprocedural urinary catheter insertion in the setting of immunosuppression, may have led to dissemination of the bacterium. Although symptom onset followed a corticosteroid injection, direct inoculation is considered less likely because *M. hominis* is not a typical skin commensal and septic arthritis after joint injection is rare [15,16]. Rather, the corticosteroid injection was performed to help alleviate pain from the unrecognized developing infection.

Diagnosis of *M. hominis* infection is often delayed because of its lack of a cell wall and slow growth in standard culture systems, which also confer intrinsic resistance to beta-lactams and glycopeptides. Unlike other Mycoplasma species such as *M. pneumoniae* that respond to macrolides such as azithromycin, *M. hominis* exhibits intrinsic resistance to 14-membered and 15-membered macrolides, including erythromycin, azithromycin, and clarithromycin, mediated by a G2057A transition in the 23S ribosomal ribonucleic acid (rRNA) gene [17,18]. Detection often requires prolonged culture incubation or molecular diagnostics such as PCR. Recovery may be improved with specialized media such as SP-4 medium, although *M. hominis* can also grow on routine agar media [19,20]. Colonies are typically tiny and may be overlooked without careful examination, and visualization of the classic fried-egg morphology generally requires low-power microscopic magnification [21]. These characteristics likely explain why only pinpoint colonies were observed in our case despite successful culture on routine media [21,22].

Treatment of invasive *M. hominis* infection generally requires both surgical source control and antimicrobial therapy. Active agents include tetracyclines, fluoroquinolones, clindamycin, and 16-membered macrolides such as josamycin [23,24]. Tetracycline resistance occurs in approximately 10–13% of isolates and is most commonly mediated by acquisition of the tetracycline resistance determinant *tet(M)* [24,25]. Because there are no standardized treatment guidelines for *M. hominis* septic arthritis, particularly in immunocompromised patients, the optimal antimicrobial regimen and duration of therapy remain uncertain [21,25]. Available evidence is limited to case reports and small case series, and susceptibility testing is often unavailable because of the specialized culture methods required [21]. In our case, susceptibility testing could not be performed because the isolate could not be successfully subcultured. Therefore, treatment with levofloxacin and doxycycline for six weeks was selected based on published experience, the known resistance profile of *M. hominis*, and the severity of infection. A recent systematic review of 65 cases of invasive *M. hominis* infection found that prolonged antimicrobial therapy and doxycycline-containing regimens, particularly in combination with fluoroquinolones, were associated with improved outcomes [26].

Clinicians should consider *M. hominis* in immunocompromised patients with culture-negative septic arthritis, particularly when purulent synovial fluid is present despite negative Gram stains and routine cultures [27]. A systematic review found that most cases of mycoplasmal septic arthritis occurred in immunocompromised hosts and emphasized considering mycoplasmal infection in patients with persistent culture-negative disease or failure to respond to beta-lactam therapy [28]. In such cases, prolonged culture incubation and molecular diagnostics, including 16S rRNA gene PCR, may facilitate diagnosis [21,22,29,30]. In our patient, *M. hominis* was identified only after extended culture incubation despite extensive purulence and initially negative cultures, highlighting the importance of maintaining suspicion for fastidious pathogens.

*M. hominis* should be recognized as a potential cause of extragenital infection in patients receiving CD20-directed monoclonal antibody therapy, including obinutuzumab. Early recognition, prolonged culture incubation when clinically indicated, and prompt initiation of appropriate antimicrobial therapy are critical to achieving favorable outcomes in this immunocompromised population.

## Abbreviations used

CLL: chronic lymphocytic leukemia

SLL: small lymphocytic lymphoma

IV: intravenous

CAN: Colistin-Nalidixic Acid

BBE: Bacteroides Bile Esculin

LKV: Laked Blood Kanamycin Vancomycin

PCR: polymerase chain reaction

rRNA: ribosomal ribonucleic acid

## CRediT authorship contribution statement

**Katherine Benandi:** Writing – review & editing, Writing – original draft, Validation, Methodology, Investigation. **Lucas S. Blanton:** Writing – review & editing, Supervision, Methodology, Conceptualization.

## Ethical Approval

IRB approval was not required for this case report.

## Consent

Written informed consent was obtained from the patient for publication of this case report and any accompanying images.

## Funding sources

None.

## Declaration of Competing Interest

The authors declare that they have no known competing financial interests or personal relationships that could have appeared to influence the work reported in this paper.
